# Crosstalk between Anxiety and Depression and Inflammatory bowel diseases: preliminary data on circulating miRNAs

**DOI:** 10.1192/j.eurpsy.2024.193

**Published:** 2024-08-27

**Authors:** I.-C. Matei, M. Dobre, E. Milanesi, T. E. Manuc

**Affiliations:** ^1^Psychiatry, Clinica Nutrimed; ^2^Victor Babes National Institute for Pathology; ^3^Gastroenterology, Fundeni Clinical Institute; ^4^Gastroenterology, University of Medicine and Pharmacy Carol Davila, Bucharest, Romania

## Abstract

**Introduction:**

Numerous studies have established a heightened prevalence of anxiety and depression (A&D) in individuals diagnosed with Inflammatory Bowel Diseases (IBD) when compared to the general population. Research indicates that patients with active IBD exhibit a higher frequency of anxiety symptoms and depression symptoms compared to those with inactive disease. In patients with IBD, anxiety was linked to reduced medication adherence and an increased likelihood of undergoing surgery. Furthermore, associations were identified between depression and an elevated risk of disease relapse, as well as a poorer response to treatment in IBD patients. In both IBD and depression, there is evidence of disruptions in circulating miRNAs.

**Objectives:**

One facet of the ongoing project titled “The brain-gut axis linking inflammatory bowel disease with anxiety and depression: the inflammation-microbiome network” (CRP/ROU21-01) involves the exploration of circulating miRNA profiles in various patient groups.

**Methods:**

These groups encompass IBD patients with symptoms of anxiety and/or depression (IBD+A&D+), patients lacking anxiety and depression symptoms (IBD+A&D-), a cohort of individuals without IBD but experiencing depressive and anxiety symptoms (IBD-A&D+), and a control group (IBD-A&D-). Thus far, our investigation has entailed screening a comprehensive panel of 179 miRNAs in the plasma of six IBD patients and 12 non-IBD patients (CTRL) to identify a subset of highly dysregulated miRNAs. MiRNA isolation was achieved using the miRNeasy Serum/Plasma Kit, and miRNA expression levels were assessed via quantitative reverse transcription-polymerase chain reaction (qRT-PCR) utilizing the Human serum/plasma focus, MIRCURY LNA miRNA Focus PCR panel (Qiagen).

**Results:**

Our statistical analysis revealed significant differential expression in 45 miRNAs (p<0.05). Specifically, we identified 29 miRNAs with elevated expression and seven miRNAs with reduced expression. Among these dysregulated miRNAs, 15 (miR-223-3p, miR-143-3p, let-7f-5p, miR-30b-5p, miR-26a-5p, let-7a-5p, miR-339-5p, let-7d-5p, miR-221-3p, miR-191-5p, let-7g-5p, miR-24-3p, miR-107, miR-26b-5p, miR-320b) were associated with depression and/or anxiety and were previously identified as dysregulated in the plasma of patients in other studies. These miRNAs will soon undergo evaluation in the plasma of IBD-A&D+ and IBD+A&D+ patients.

**Conclusions:**

These initial findings provide us with a panel of circulating miRNAs that warrant further investigation in the aforementioned patient groups. The miRNA profile we obtained may either be unique to IBD or linked to the intricate phenotypes of IBD occurring concurrently with anxiety and depression. A more profound comprehension of these mechanisms will aid in the development of enhanced

diagnostic tools and disease monitoring strategies, as well as the exploration of innovative therapeutic approaches.

**Disclosure of Interest:**

None Declared

